# Enhancing Teacher Training Through Self‐Efficacy and Emotional Intelligence: A Conditional Process Model of Pre‐Service Teachers’ Well‐Being and Academic Achievements

**DOI:** 10.1002/brb3.70578

**Published:** 2025-05-26

**Authors:** Frank Quansah, Daniel William Essel, Nancy Asieduwaa Gyapong, Isaac N‐Nandi Yabana, Yayra Dzakadzie, Patrick Asamoah, Kingsley Addison

**Affiliations:** ^1^ Department of Educational Foundations University of Education Winneba Ghana; ^2^ The National Council for Curriculum and Assessment, NaCCA Accra Ghana

**Keywords:** depression, emotional intelligent, mental health, moderated mediation, pre‐service teachers, self‐efficacy

## Abstract

**Introduction:**

Extant literature has established the relationship between psychological well‐being (PWB) and academic achievement (CGPA). Yet, little is known about the intervening roles of key variables such as emotional intelligence (EI) and academic self‐efficacy (ASE). This study investigates the complex roles of pre‐service teachers’ EI and ASE in the relationship between PWB and CGPA.

**Methods:**

Using a cross‐sectional design, data from 660 third‐ and final‐year pre‐service teachers at the University of Education, Winneba, were analyzed. The study assessed participans PWB, ASE, EI, and CGPA, employing validated scales and controlling for age and sex as covariates.

**Results:**

The findings revealed that higher PWB, ASE, and EI significantly predict better CGPA. However, ASE emerged as a strong mediator in the PWB‐CGPA relationship, with a direct and consistent influence across varying levels of EI. Notably, EI significantly moderated the effect of PWB on CGPA, suggesting its buffering role against academic stress and its ability to enhance academic outcomes.

**Conclusion:**

The study concludes that interventions aimed at enhancing ASE could provide the most substantial benefits for academic achievement, regardless of students' EI levels. These findings underscore the importance of integrated teacher education programmes that foster ASE and EI, thus equipping pre‐service teachers with the tools needed to navigate academic challenges and excel professionally.

## Graphical abstract

Psychological well‐ being significantly boosts academic achievements among pre‐ service teachers, especially when supported by strong academic self‐ efficacy. Emotional intelligence enhances this relationship, highlighting the need to develop both confidence and emotional skills to foster academic success in teacher education.

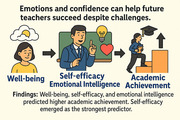

## 1 Introduction

Amidst the numerous functions of academic institutions, one of the key roles that stakeholders (including school heads, parents, teachers, and students) look up to is improving academic achievements of learners. In this regard, students’ academic achievements have become a crucial indicator of institutional reputation and ranking in both first‐ and second‐cycle institutions as well as the higher education institutions (Owusu‐Boadu et al. 2021). This issue also plays an important role in building the human resource capital of a country, which could boost the country's economic and social development (Ali et al. 2009). Therefore, teachers and educators continuously make efforts to adopt various teaching approaches and strategies to improve and sustain students’ academic achievements; yet not all students perform satisfactorily.

Though academic achievement has been strongly linked to intelligence quotients (IQ), recent studies have indicated that IQ alone is not an adequate predictor of students’ academic success. Mohzan et al. (2013), for example, reported that only 20 percent of a person's success can be linked to their IQ. The remaining 80 percent has been largely attributed to key factors such as socio‐economic status, psychological well‐being (PWB), study process, student engagement, self‐efficacy, stress and emotional components, age, sex, nationality, and marital status (see Ansah et al. 2020; Aydin 2017; Morales‐Rodríguez et al. 2020; Roksa and Kinsley 2019; Tannoubi et al. 2023). Among these factors, the well‐being of students has been identified as critical, taking the centre of discussion in recent literature on academic achievements. This conversation is sparked by the increased prevalence of depression and stress experiences of university students (Adu Henaku et al. 2024; Faisal et al. 2022; Quansah et al. 2022; Sheldon et al. 2021). Evidence from a systematic review supported this observation that mental health and well‐being challenges were common among undergraduate students with high suicidal ideation of about 21 percent prevalence (Sheldon et al. 2021).

### 1.1 PWB and Academic Achievement

The relationship between well‐being and academic achievement has been widely explored among students (particularly at the higher education level) in the literature, with the studies yielding mixed findings. While some scholars found a negative or no relationship between students’ well‐being and academic achievements (see Amholt et al. 2020; Klapp et al. 2024), the overwhelming proportion of the studies revealed a positive association (see Bukhari and Khanam 2017; Cárdenas et al. 2022; Howell 2009; N‐yelbi and Awuku‐Larbi 2024; Kaya and Erdem 2021; Mustafa et al. 2020; Turashvili and Japaridze 2012; Zefi et al. 2022). Howell (2009), for example, identified three well‐being profiles (i.e., flourishing, moderately mentally healthy, and languishing) and found that students in the flourishing well‐being group reported higher self‐control and academic achievements. Furthermore, findings from Cárdenas et al.'s (2022) study highlighted not only the short‐term impact of students’ well‐being on academic achievement but also its long‐term effect, stressing that students with positive well‐being were more likely to perform excellently, even after 7 months. It is important to add that Cárdenas and colleagues’ findings emerged after controlling for key confounding variables, including previous test scores, age, and sex.

In a meta‐analysis research, a significant positive relationship was also revealed between students’ well‐being and academic achievements, with a small effect size (Kaya and Erdem 2021). The authors further discovered that the effect size of the well‐being‐achievement relationship significantly varied across the ages of participants. For instance, the relationship between well‐being and academic achievement was stronger for younger students and weaker for older students. Interestingly, Amholt et al. (2020) in their review also identified that studies which involved younger students found a significant positive relationship between well‐being and academic achievement, but this relationship was non‐significant for older students. In the Ghanaian context, N‐yelbi and Awuku‐Larbi (2024) also established a strong positive relationship between university students’ well‐being and academic achievements, with sex variations in the variables observed.

The literature is dominated by the findings that students with positive well‐being are more likely to demonstrate better academic achievement compared to those with negative well‐being. The contrary findings presented by other studies (i.e., no relationship or negative relationship) could be attributed to differences in the contextual variables in those studies. A key takeaway from the well‐being‐achievement relationship is the presence of some confounding variables that potentially cause a varying magnitude of the impact of students’ well‐being on their academic achievements. Notably, sex and age were found as important confounding variables that are consistent across the previous studies (Cárdenas et al. 2022; Kaya and Erdem 2021; N‐yelbi and Awuku‐Larbi 2024). This study takes clues from previous literature, thereby using sex and age as covariates to partial out the effect of these variables in the relationships examined.

### 1.2 The Roles of Academic Self‐Efficacy (ASE) and Emotional Intelligence (EI) in the Link Between Students’ Well‐Being and Academic Achievement

Given that higher education students, especially in Africa, experience mental health problems due to heavy academic workload, the pressure of maintaining good grades, and limited financial resources, among others (Commey‐Mintah et al. 2022; Quansah et al. 2023; Quansah et al. 2022), there is a greater need to understand how these mental health issues could be addressed. As earlier discussed, these mental health concerns, when not addressed, negatively impact the academic achievementof the students. Extant literature has identified that the two key protective variables that serve as a buffer against students’ mental health problems (or negative well‐being) are ASE (García‐Álvarez et al. 2021; Gulley et al. 2021; Wen et al. 2021) and EI (Hussien et al. 2020; Bermejo‐Martins et al. 2021; García‐Álvarez et al. 2021). These studies have revealed that ASE and EI are positively related to students’ well‐being.

In more detail, Kristensen et al. (2023) study discovered that ASE partially mediates the impact of academic stress on psychological distress, indicating the role of well‐being in fostering students’ confidence in their academic abilities. The authors indicated that students’ well‐being significantly predicted their ASE, thereby determining their state of distress. Similarly, Liu et al. (2024) explored the longitudinal relationships between stress and ASE among students at elite colleges in China. Their findings indicated that higher stress levels negatively impact ASE, suggesting that maintaining positive PWB is essential for fostering ASE. Furthermore, Iqbal et al. (2021) also found that EI interacts with the mental health concerns of students and significantly contributes to fostering psychoeducational development among university students. It is in the same vein that Bartos et al. (2021) reiterated that EI promotes the development of resilience and acts as a coping resource for mental health crises.

In other studies, it has been found that students with low EI often experience higher mental health challenges, which negatively impact their ASE (Shengyao et al. 2024). This observation emphasizes the positive relationship existing between EI and ASE, which further impacts well‐being. Bandura's self‐efficacy theory supports how EI can enhance self‐efficacy by improving emotional regulation, stress management, and coping strategies (Bandura 1977; Bhati and Sethy 2022). Relatedly, students with higher EI exhibited greater ASE, which in turn improved their academic achievements (Baños et al. 2023; MacCann et al. 2020). In other words, the student's ability to manage emotions effectively allows him/her to cope with academic demands, maintain motivation, and persist in the face of challenges. It is, therefore, not surprising that systematic literature reviews consistently identified self‐efficacy as the strongest and most consistent predictor of university students’ academic achievements (Bartimote‐Aufflick et al. 2016; Yokoyama 2019).

Even though no study provides insight into the explicit roles of EI and ASE in the link between students’ well‐being and academic achievements, the self‐determination theory offers a theoretical lens for understanding the nuanced roles played by the two intervening variables (i.e., ASE and EI). The self‐determination theory reiterates that individuals with satisfied psychological needs of autonomous, competence, and a sense of relatedness are more motivated to perform academic tasks well (Deci and Ryan 2012). In the context of this research, the theory supports the view that students who have control over their learning and are well confident in handling academic tasks (i.e., ASE) are more likely to perform well. Similarly, students who feel they belong in the school setting, are connected, valued, and supported by mentors/colleagues (i.e., EI) during practicals/internships are more likely to demonstrate excellent performance. Within the self‐determination theory, these variables interact and create a sense of motivation, providing a clear context for positive well‐being (Axford et al. 2014) and subsequently leading to superior academic achievements. Although the relationships among the key variables of the theory are dynamic (Kleinkorres et al. 2020), existing literature reviews are more oriented towards the notion that positive well‐being indirectly impacts academic achievements through ASE and is moderated by EI.

### 1.3 The Teacher Training Context

The study was conducted in the teacher training context, recruiting pre‐service teachers undergoing training at the University of Education Winneba (UEW) in Ghana, which generally has a male‐female distribution of about 55%–45%. The teacher training process at the university level in Ghana generally takes 4 years to obtain a Bachelor's degree in education, although this duration may differ based on entry level (Quainoo et al. 2020). The programme is structured as an intensive experiential training (more intense at the third and fourth year levels) which requires teaching practices and taught courses culminating between 120 and 140 credits with students taking up to 21 hours of contact classes per week (Quansah and Ankoma‐Sey 2020). With the new curriculum reforms in the educational space, students participate in supported teaching in school (STS), where they visit selected schools concurrently throughout their training to attain practical, school‐based learning experiences (National Council for Curriculum and Assessment [NaCCA] 2019).

While there are giant efforts from the teacher preparation institutions and the government in improving and sustaining quality teacher education, several challenges are present, including a high lecturer‐student ratio, inadequate teaching infrastructure and resources, insufficient teachers meeting the training needs of students, high academic workload, and financial constraints, among others (Aboagye and Yawson 2020; Buabeng et al. 2020; Quansah et al. 2023). These challenges, coupled with the intensive nature of the teacher education programme, appear to have resulted in mental health consequences for these students. In fact, studies conducted in the teacher education institution (selected for this study, UEW) revealed negative well‐being experiences of the teacher‐trainees (Quansah et al. 2022; Quansah et al. 2024). Meanwhile, there is evidence to support that not all students in this institution (i.e., UEW) are able to graduate as expected (Ibrahim et al. 2022). In addition, recent headlines confirmed that some pre‐service teachers in the same institution had cumulative grade point average (CGPA) less than 1.0 and were on the verge of being dismissed (Agyei‐Lartey 2025).

Although there is anecdotal evidence from the authors to the fact that the low academic achievement among the pre‐service teachers in UEW is largely attributed to the high burdens and challenges they experienced, there is clearly no existing literature in this regard, especially in developing nations like Ghana. We, however, argue that the EI and ASE of the pre‐service teachers could make a difference in how the negative well‐being experiences could impact their academic achievements (i.e., CGPA is used as the indicator in this study). This is critical as pre‐service teachers’ EI defines how emotions experienced are perceived, understood, and managed, thereby equipping them to cope with academic stress and interpersonal demands in their training (Turner and Stough 2020). Similarly, pre‐service teachers need to build their belief in their ability to academically succeed, further enhancing their goal‐setting, persistence, motivation, and learning strategies (Kula and Taşdemir 2014). Transiting from a pre‐service teacher to an in‐service teacher is academically and emotionally daunting, and thus, teacher‐trainees must have the capacity to manage their learning and start behaving like educators (Owusu‐Agyeman and Amoakohene 2020). Exploring the roles of these variables in the PWB‐achievement relationship is critical for informing policy and interventions aimed at enhancing teacher preparation programmes.

**FIGURE 1:**
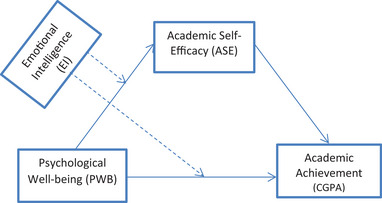
Conceptual framework showing the moderated mediation effect of **EI** and **ASE** in the relationship between **PWB** and **CGPA**. Source: Adopted from Hayes’ (2018) Conditional Process Model 8.

Based on the afore‐discussed literature‐based issues, we proposed two hypotheses which guided the development of a conceptual framework, subsequently (see Figure 1):

$H_{A}$1: Pre‐service teachers’ EI will significantly moderate the relationship between PWB and (a) CGPA and (b) ASE.
$H_{A}$2: Pre‐service teachers’ ASE will significantly mediate the relationship between PWB and CGPA as moderated by their EI.

The conceptual framework was designed in line with the conditional process modelling strategy (Model 8) proposed by Hayes (2018), combining both mediation (solid lines) and moderation (broken lines) analyses. Moreover, the framework is also a reflection of the understanding from previous studies and related theories (i.e., self‐determination and self‐efficacy theories) regarding the intersecting roles of PWB, EI, ASE, and CGPA—the ASE transmits the effect of PWB on CGPA with EI functioning as a moderator of the indirect effect of ASE as well as the direct effect of PWB on CGPA. The conceptual framework reflects a theory‐driven structure that is consistent with the analytical strategy in the study.

## 2 Methods

### 2.1 Participants

This study was conducted using a cross‐sectional design. Data were collected from 660 pre‐service teachers in the regular stream mode at the University of Education, Winneba (UEW), Ghana. The study focused on only third‐ and final‐year students based on notable methodological and conceptual considerations. First, the CGPA in the early years of the pre‐service teachers’ training is usually unstable and may not reflect long‐term academic achievement profiles as a result of transitional factors (Cheong and Ong 2014). In the third‐ and final‐year period, the CGPA becomes more stable and relatively representative of the students’ overall training and achievements. Secondly, during the third‐ and fourth‐year period of the pre‐service teachers’ training, their PWB, EI, and ASE would have become fully developed and less volatile (Agormedah et al. 2022; Maheshwari and Gujral 2021). Typically, the third‐ and fourth‐year pre‐service teachers have had greater exposure to academic challenges, experiential teaching experiences, reflective practices, and initiation of pedagogical practices—factors that meaningfully shape PWB, EI, and ASE. Based on these considerations, focusing on the third‐ and fourth‐year pre‐service teachers is critical for ensuring that the measured indicators are contextually appropriate and developmentally relevant to the study context.

The participants for the study stratified sampling technique, with the faculty level being the starting point and the departments being the end‐point of the stratification. The final sample was obtained from the departmental level, and these individuals were contacted to confirm their participation consent via initial text messages sent to them. All ethical insights were explained to the participants through the initial message before they could provide their consent. Initially, 1000 messages were sent to the potential participants, with only 814 of them confirming their willingness to participate. We further sent the online survey to the 814 pre‐service teachers, and they were given a period of three days to respond. After some follow‐up messages, only 660 responded to the survey. This final sample included 417 males (63.2%) and 243 females (36.8%). The participants’ ages ranged from 19 to 36 years, with a mean age of 28.32 years (SD = 0.439). This age distribution reflects the diverse entry pathways into the teacher education programme. In particular, some regular pre‐service teachers are admitted through the post‐diploma route, having previously completed a three‐year Diploma in Education, and are now pursuing a Bachelor's degree in Education. Many of these individuals typically enroll on their respective programmes in their 30s, which accounts for the relatively high mean and maximum ages observed in the sample.

## 3 Measures

### 3.1 Predictor Variable: PWB

PWB was measured using Ryff's Psychological Well‐Being Scale (Ryff et al. 2010). The scale has 18 items with six dimensions, namely: autonomy, environmental mastery, personal growth, positive relations with others, purpose in life, and self‐acceptance. Each dimension had three items scored on a 7‐point Likert scale (1 = strongly agree, 2 = somewhat agree, 3 = a little, 4 = neither agree nor disagree, 5 = a little disagree, 6 = somewhat disagree, 7 = strongly disagree) of which 10 of the items were reversed coded. Some of the questions on the scale include:’ *I tend to be influenced by people with strong* opinions’ (autonomy); ‘*The demands of everyday life often get me down*’ (environmental mastery); ‘*For me, life has been a continuous process of learning, changing, and growth’* (personal growth); ‘*Maintaining close relationships has been difficult and frustrating for me*’ (positive relations with others); ‘*Some people wander aimlessly through life, but I am not one of them*’ (purpose in life); ‘*I like most parts of my personality*’ (self‐acceptance). Previous vaidation research has revealed that a second‐order factor structure of the PWB scale produces higher measurement precision (Abbott et al. 2010). A higher score on the scale represents positive well‐being or greater levels of well‐being, whereas a lower score reflects negative well‐being or lower levels of well‐being. The scale recorded sufficient Cronbach's alpha estimates for each dimension: autonomy (0.70), environmental mastery (0.75), personal growth (0.74), positive relations with others (0.78), purpose in life (0.75), and self‐acceptance (0.80). In this study, the Cronbach's alpha reliability coefficients ranged from 0.74 to 0.81 across the dimensions. The coefficients were deemed sufficient (Pallant 2020).

### 3.2 Outcome Variable: Academic Achievement (CGPA)

The academic achievement of the pre‐service teachers was measured using the cumulative grade point average (CGPA). The CGPA is a widely used, valid, standardized, and institutionally verified metric of academic achievement of students across multiple academic semesters in the higher education space (Richardson et al. 2012). While academic achievement in specific subject areas may provide key insights, CGPA offers a more stable and comprehensive measure of students’ long‐term academic achievements and engagements. The CGPA indicator has been found suitable for statistical modelling of psychological traits such as PWB, ASE, and EI, which usually impact overall academic achievement and functioning rather than specific domain outcomes (Richardson et al. 2012).

Participants were encouraged to report their CGPA from the students’ online portal during data collection and self‐reporting on the questionnaire. The participants who consented to respond to their survey were asked to upload a screenshot of their CGPA from their student portal with their names and index numbers. We assured the participants that these data were used for academic purposes only and thus, no identifier would be used for the data analytical processing. The CGPA ranges from 0 to 4.0, with a higher CGPA showing better academic achievement and a lower CGPA depicting poor academic achievement.

### 3.3 Mediator Variable: ASE

Students’ ASE in this study was to inquire how students believe in their ability to successfully perform and accomplish academic tasks. This construct was measured using the General Academic Self‐Efficacy Scale, which is a unidimensional scale (GASE: Nielsen et al. 2018). The scale comprises five (5) items on a 5‐point Likert scale (1 = strongly disagree, 2 = disagree, 3 = Neutral, 4 = agree, and 5 = strongly agree). A sample item on the scale is: “*I generally manage to solve difficult academic problems if I try hard enough*”. Higher scores on the GASE represent better ASE and vice versa. The psychometric properties of the GASE have been established with a Cronbach's alpha reliability of 0.81 (Akanni and Oduaran 2018). Using the Cronbach's alpha estimation method, a reliability coefficient of 0.79 was obtained in this study and thus, considered sufficient (Pallant 2020).

### 3.4 Moderator Variable: EI

In the quest to know how students can recognize, understand, manage, and influence their own emotions and the emotions of others, the Brief Emotional Intelligence scale (BEIS‐10) with 10 items on a 5‐point Likert scale developed by Davis and colleagues in 2010 was used to measure students’ EI (Davies et al. 2010). The scale had five dimensions: appraisal of own emotions, appraisal of others’ emotions, regulation of own emotions, regulation of others’ emotions, and utilization of emotions with two items each. With response options from 1 = “strongly disagree” to 5 = “strongly agree”. Some typical items on the scale include ’ *I know why my emotions change’* (appraisal of own emotions,); ‘*I can tell how people are feeling by listening to the tone of their voice*’ (appraisal of others’ emotions,); ‘*I seek out activities that make me happy’*(regulation of own emotions); ‘*I arrange events others enjoy’*(regulation of other's emotions) and ‘*When I am in a positive mood, I am able to come up with new ideas*’(utilization of emotions). An overall Cronbach alpha coefficient value of 0.89 was obtained for the scale with alpha values of 0.80, 0.82, 0.80, 0.83, and 0.75, respectively, for the domains of appraisal of own emotions, appraisal of others’ emotions, regulation of own emotions, regulation of other's emotions and utilization of emotions. This study recorded Cronbach alpha reliability coefficients ranging from 0.72 to 0.79—these coefficients are considered sufficient (Pallant 2020). Higher scores on the BEIS‐10 scale suggest sufficient EI, whereas a lower score denotes otherwise. Recent validation research identified the EI latent structure as a second‐order factor and thus confirmed the scale's psychometric properties and items’ usefulness (Yahyaoui et al. 2025).

### 3.5 Covariates: Sex and Age

Sex and age were used as the covariates in the study. Sex was conceptualized as male and female, whereas age was measured on a continuous basis. Age and sex were included in this study based on documented evidence from previous studies, which have shown that these two variables influence one or a combination of PWB, ASE, EI, and CGPA (Agormedah et al. 2022; Chen and Cheng 2023; Costa et al. 2021; García‐Álvarez et al. 2021). Moreover, students enter the pre‐service teacher training programme via different routes, resulting in a wider age range than typically observed in higher education levels. This variability can impact exposure to academic experiences, thereby shaping PWB, EI, and ASE (Boakye and Ampiah 2017). In general, the decision to include sex and age as covariates is in the light of reducing the confounding effect of the relationships tested, thereby reducing omitted variable biases and improving the precision and validity of the results.

### 3.6 Statistical Analyses

Data was screened and edited to check for outliers and normality. Descriptive statistics such as frequencies, percentages, mean, standard deviation, skewness, and kurtosis were performed to present the demographic variables and explore the key variables. Again, Pearson product moment correlation was conducted to understand the associations among the variables. The moderated mediation analysis was used as the statistical procedure to test the hypotheses in this study using Hayes’ PROCESS model 8 in the SPSS software (Barnet‐Verzat and Wolff 2008) with 10,000 bootstrap samples. A conditional process analysis, in this context, means that the indirect link between a predictor (PWB) and an outcome (CGPA) is influenced by a mediator (ASE) at varying levels of a moderator (EI) (Hayes 2018). In Model 8, the adequacy of EI moderating the relationships between (a) PWB and ASE, and (b) PWB and CGPA are tested. In all, the moderated mediated seeks to find the effect of PWB on CGPA through the mediator ASE at different levels of EI. The analysis was conducted with a 95% confidence interval and a significance level of 0.05. Significance was determined by checking if the confidence interval for the parameter excluded zero. The conditional indirect effect was assessed using bootstrapping with 10,000 samples, which improves the accuracy of parameter estimates by offering a straightforward method for estimating confidence intervals and standard errors. Given that the multidimensional constructs (PWB and EI) had a second‐order structure, the total scores from the scale, instead of scores from sub‐domains, were used for the major analyses in the study.

## 4 Results

### 4.1 Preliminary Analysis

The linear relationships existing among the variables and other descriptive statistics for the variables were explored. The output from Table 1 reveals that there are both positive and negative interrelationships among the four variables. These associations ranged from −0.09 to 0.750. For instance, PWB was positively associated with ASE (*r* = 0.183) and CGPA (*r* = 0.512) but negatively associated with EI (*r* = −0.097). Similarly, ASE was also negatively correlated with EI (*r* = −0.325). Further, PWB showed strong positive correlations with personal growth (PGRO) (*r* = 0.750; *r* = 0.750), positive relations (PREL) (*r* = 0.624; *r* = 0.624), and self‐acceptance (SE‐AC) (*r* = 0.621; *r* = 0.621) (see Table 1).

**TABLE 1:** Descriptive statistics of the key variables.

	AUT	MAS	PGRO	PREL	PLIFE	SE‐AC	PWB	EI	ASE	CGPA
Autonomy (AUT)	1									
Environmental mastery (MAS)	0.226^**^	1								
Personal growth (PGRO)	0.444^**^	0.285^**^	1							
Positive relation (PREL)	0.218^**^	0.098	0.355^**^	1						
Purpose of life (PLIFE)	0.077	0.089	0.257^**^	0.275^**^	1					
Self‐acceptance (SE‐AC)	0.131	0.349^**^	0.367^**^	0.321^**^	0.199^**^	1				
Psychological well‐being (PWB)	0.577^**^	0.539^**^	0.750^**^	0.624^**^	0.540^**^	0.621^**^	1			
Emotional intelligence (EI)	−0.173^*^	0.010	−0.048	−0.082	0.033	−0.105	−0.097	1		
Academic self‐efficacy (ASE)	0.101	0.110	0.125	0.147^*^	0.034	0.175^**^	0.183^**^	−0.325^**^	1	
CGPA	0.105	0.202^**^	0.281^**^	0.215^**^	0.240^**^	0.130	0.324^**^	0.472^**^	0.512^**^	1
Variable range	1–7	1–7	1–7	1–7	1–7	1–5	1–5	1–5	1–5	0–4
Mean	5.14	4.91	5.54	4.54	4.80	5.65	5.10	2.21	3.78	2.04
Standard deviation	1.31	1.19	1.34	1.13	1.38	1.11	0.77	.96	1.22	0.54
Skewness	−0.363	−0.163	−0.170	−0.145	−0.284	−0.468	−0.127	1.22	−1.15	1.049
Minimum	1	1.67	1	1	1	2.33	3.11	1	1	1.16
Maximum	7	8.33	7	7	7.33	7	6.61	5	5	3.87

The PWB had a mean of 5.10 and a standard deviation of 0.77. Mean values of 2.21 and 3.78 were obtained for the ASE and CGPA variables, respectively (see Table 1). All the skewness (±2) and kurtosis (±7) values were acceptable and depicted that the residuals for the responses on each variable were normally distributed (Tabachnick and Fidell 2013).

### 4.2 Moderating the Role of EI in the Relationship Between PWB and (a) ASE, and (b) Academic Achievement (CGPA)

As presented in Table 2, pre‐service teachers’ EI did not significantly moderate the relationship between PWB and ASE, (B = 0.104, BootSE = 0.0637, BootCI [−0.0207, 0.2293]), suggesting that the effect of PWB on ASE does not significantly vary based on different levels of EI. In the same model, PWB had a significant positive effect on ASE (B = 0.262, BootSE = 0.059, BootCI [0.147, 0.378]), suggesting that higher PWB predicted improved ASE.

**TABLE 2:** Moderation effect of EI on the relationship between PWB and (a) AA and (b) ASE.

Outcome Variable	Model Summary	Predictor	B	SE	*t*‐value	*p*‐value	95% BootCI (LLCI—ULCI)
Academic self‐efficacy (ASE)	*R* = 0.366	Constant	3.983	0.2014	19.7807	<0.0001	3.588, 4.379
	*R* ^2^ = 0.134	PWB	0.262	0.059	4.462	<0.001	0.147, 0.378
		EI	−0.383	0.046	−8.321	<0.001	−0.474, −0.293
		Interaction (PWB x EI)	0.104	0.064	1.638	0.102	−0.021, 0.229
		Male (Female‐Ref)	−0.086	0.097	−0.887	0.375	−0.277, 0.105
		Age	−0.016	0.049	−0.322	0.747	−0.113, 0.081
Cumulative GPA (CGPA)	*R* = 0.890	Constant	0.956	0.055	17.147	<0.001	0.847, 1.066
	*R* ^2^ = 0.792	PWB	0.198	0.013	15.141	<0.001	0.172, 0.224
		ASE	0.310	0.009	36.194	<0.001	0.293, 0.327
		EI	0.410	0.011	38.625	<0.001	0.389, 0.431
		Interaction (PWB x EI)	0.057	0.0140	4.074	<0.001	0.030, 0.084
		Male (Female‐Ref)	−0.023	0.015	−1.551	0.120	−0.040, 0.001
		Age	−0.020	0.023	−0.761	0.450	−0.060, 0.030

Abbreviations: ASE, academic self‐efficacy; BootCI, bootstrap confidence interval; EI, emotional intelligence; LLCI, lower limit confidence interval; PWB, psychological well‐being; ULCI, upper limit confidence interval.

Surprisingly, EI was found to be negatively associated with ASE (B = −0.383, BootSE = 0.046, BootCI [−0.474, −0.293]). Although this inverse association between EI and ASE was not hypothesized, it may represent context‐specific dynamics within the Ghanaian pre‐service teacher training context and the findings, therefore, call for a more explorative investigation. We have, however, extensively discussed this finding, subsequently in this research report.

Further results from Table 2 revealed a significant moderation effect of EI on the relationship between PWB and CGPA (B = 0.057, BootSE = 0.014, BootCI [0.030, 0.084]), suggesting that the relationship between PWB and CGPA varies depending on the levels of EI. In addition, PWB also significantly predicted CGPA (B = 0.1979, BootSE = 0.0131, BootCI [0.1722, 0.2236]). Besides, ASE significantly predicted CGPA (B = 0.3100, BootSE = 0.0086, BootCI [0.2932, 0.3269]) by confirming the role self‐efficacy plays in academic success. EI also positively predicted CGPA (B = 0.4098, BootSE = 0.0106, BootCI [0.3890, 0.4306]), implying that the higher the EI, the better the CGPA.

### 4.3 Moderated Mediated Effect of EI and ASE in the Relationship Between PWB and Academic Achievement (CGPA)

The study also hypothesized that ASE will significantly mediate the relationship between PWB and CGPA as moderated by the EI. The analysis showed a significant positive direct effect of PWB on CGPA at different levels of EI: low EI (B = 0.143, BootSE = 0.018, BootCI [0.108, 0.178]), moderate EI (B = 0.198, BootSE = 0.013, BootCI [0.172, 0.224]), and high EI (B = 0.253, BootSE = 0.020, BootCI [0.214, 0.292]) (see Table 3). Moreover, the indirect effect of the mediating variable ASE in the association between PWB and CGPA for pre‐service teachers with low, moderate, or high EI was significant and stronger at each EI level. On the contrary, the index of moderated mediation (B = 0.03, CI [−0.01, 0.08]) was not significant at a 95 percent confidence level. Hence, the mediated effect of PWB on CGPA through ASE strengthens at higher levels of EI, but this interaction does not reach significance for moderated mediation.

**TABLE 3:** Conditional Direct and Indirect Effects of EI and ASE on PWB and AP.

Effect type	EI levels	*β*	SE	95% CI (LLCI—ULCI)
**Conditional direct effects**	low	0.143	0.018	0.108, 0.178
	Moderate	0.198	0.013	0.172, 0.224
	High	0.253	0.020	0.214, 0.292
**Conditional indirect effects**	Low	0.050	0.021	0.009, 0.091
	Moderate	0.081	0.016	0.049, 0.113
	High	0.113	0.032	0.047, 0.174
**Index of moderated mediation**		0.032	0.022	[−0.011, 0.0754]

### 4.4 The Effect of the Covariates

The covariates were generally found as non‐significant predictors of the criterion variables in all the models (see Table 2). For example, age did not significantly predict ASE (B = −0.016, BootSE = 0.049, BootCI [−0.113, 0.081]) and CGPA (B = −0.020, BootSE = 0.023, BootCI [−0.060, 0.030]). In addition, sex also failed to significantly predict ASE (B = −0.086, BootSE = 0.079, BootCI [−0.277, 0.105]) and CGPA (B = −0.023, BootSE = 0.015, BootCI [−0.040, 0.001]).

## 5 Discussions

This research assessed the complex intersection of PWB and CGPA of pre‐service teachers in Ghana, taking into consideration their ASE and EI. Initial findings revealed that pre‐service teachers with higher or better PWB, ASE, and EI demonstrated higher academic achievements, as has been confirmed in earlier studies (Kristensen et al. 2023; Sun and Lyu 2022). In addition, it was observed that pre‐service teachers with better PWB exhibited higher EI, suggesting how PWB can build self‐awareness, psychological resilience, and positive relationships leading to strong EI (Halimi et al. 2021; Shengyao et al. 2024). The initial finding also revealed a counterintuitive outcome where EI negatively predicted ASE among the pre‐service teachers. Historically, a large amount of research has revealed a positive prediction of EI on ASE in diverse populations (see Baños et al. 2023; MacCann et al. 2020; Shengyao et al. 2024). This pre‐existing positive relationship has also had strong theoretical backing from Bandura's self‐efficacy theory, which highlights how strong EI enhances ASE (Bandura 1977; Bhati and Sethy 2022).

The counterintuitive finding that pre‐service teachers’ EI negatively predicts ASE could be attributed to some noteworthy reasons. First, pre‐service teachers with strong EI are normally oversensitive to emotional nuances, which usually results in over‐analysis of situations (when not managed well), leaving them in doubt about what they can do. Moreover, strong EI could translate into the use of heightened emotional coping strategies and less active coping strategies when tackling academic‐related challenges in their educational journey. Other key variables that could explain this negative EI‐ASE relationship include context‐specific challenges faced by the pre‐service teachers and culturally related expectations. Earlier discussion highlighted the significant challenges faced by pre‐service teachers in Ghana (Owusu‐Agyeman and Amoakohene 2020), and thus, those with strong EI may feel overwhelmed through empathetic behaviours leading to low confidence. With regards to culture, the collectivist culture predominance may predispose those with strong EI to prioritise relational obligation and social harmony over personal confidence, thereby reducing their ASE. This finding calls for training programmes that emphasize a balance between EI and academic training. There is a need for targeted support for pre‐service teachers with high EI that strengthens their efficacy towards handling academic‐related tasks. This counterintuitive finding also calls for further research investigation, focusing on the nuanced exploration of how EI manifests in the teacher preparation setting in Ghana and Africa, at large.

Investigating further the complex intersection of the variables, we hypothesized that pre‐service teachers’ EI will significantly moderate the PWB‐ASE and PWB‐CGPA relationships. It was revealed that the relationship between PWB and ASE did not change at different levels of EI. The EI of pre‐service might not have reached the optimum level to effectively impact the PWB‐ASE association, especially when they exhibited low EI. Notwithstanding, EI significantly moderated the relationship between PWB and CGPA. At higher levels of EI, the association between PWB and CGPA becomes stronger. Although very little is known, the few studies that have investigated EI have confirmed its essential role in enhancing well‐being and academic achievement (MacCann et al. 2020; Parker et al. 2004). In fact, higher EI serves as a buffer in managing stress and negative emotions, which further interacts with their well‐being to directly contribute to academic success (Baños et al. 2023; Shengyao et al. 2024). This finding has implications for educational institutions to design teacher education programmes that focus on offering interventions for EI development while promoting well‐being. Teaching practice sessions should prioritize the integration of EI development that translates into emotional and academic success. The findings call for stakeholders in education to consider the integration of EI‐focused mentorship training in teacher preparation institutions.

The findings on the complex intersection of the variables further reported that at low, moderate, and high EI levels of the pre‐service teachers, the indirect effect of ASE in the PWB‐CGPA relationship becomes stronger, but this interaction failed to attain significance for moderated mediation. Stated differently, the indirect effect of ASE in the relationship between PWB and CGPA is similar across the different levels of EI. We must highlight that the non‐significant moderation mediation effect does not belittle the role of PWB, ASE, and EI in impacting academic achievement (CGPA) but instead offers clarity that the effect of ASE is stable over varying EI levels. This finding suggests that emphasizing PWB and ASE interventions could benefit the academic achievement of the pre‐service teachers without tailoring interventions to EI levels. Even though EI serves as a buffer to reduce the negative impact of PWB on CGPA, focused interventions to enhance ASE would similarly benefit individuals with different levels of EI.

The general import of the findings from this study is that although better PWB and EI are relevant to enhance academic achievement (i.e., CGPA), ASE is a stronger positive driver of CGPA, since it served as a mediator and also demonstrated a higher impact across conditions. This general understanding of this finding reflects several implications. To start with, pre‐service teachers with strong ASE have higher chances of engaging in behaviours such as setting realistic goals, progress monitoring, and adopting effective learning behaviours, which become a pointer to improved CGPA compared to EI and PWB. In addition, ASE offers resilience and coping mechanism against academic‐related stressors experienced by pre‐service teachers (Kristensen et al. 2023; Liu et al. 2024). The important role of ASE in this study can also be attributed to the direct relevance of the variable to academic achievement. We note that ASE is task‐specific and directionally related to CGPA rather than EI and PWB, which are more general constructs. In this sense, the pre‐service teachers with high confidence in their ability to successfully carry out academic‐related tasks (i.e., high ASE) exhibit a high level of effort and persistence in their academics, as reflected in their CGPA. It can, therefore, be mentioned that ASE serves as the mechanism through which general traits such as PWB and EI exert their impact. These reflections from the findings on the role of ASE are strongly supported by Bandura's theory of self‐efficacy (Bandura 1977; Bhati and Sethy 2022).

The conditional process analysis outcome regarding the interplay among PWB, ASE, EI, and CGPA appears consistent with the ideology of the self‐determination theory, which stipulates that the PWB arises from satisfying autonomy, competence, and relatedness needs of pre‐service teachers (Deci and Ryan 2012). The finding that ASE stably mediated the PWB‐CGPA link irrespective of the EI levels indicates that the competence beliefs are consistent and pivotal in improving CGPA. Besides, the non‐significance of the moderated mediation outcome suggests that, even though EI is relevant, the driving effect of ASE remains robust across distinct EI profiles, stressing the essence of competence in the teacher training contexts.

The covariates also contribute to the discussion, offering insights into how interventions can be guided by their effects. Findings from the control variables showed that age and sex did not significantly predict ASE and CGPA. This finding suggests the design of a universal programme design that is more inclusive regardless of sex and age. Similarly, the non‐significance of age and sex on ASE and CGPA also reflects similar self‐efficacy and academic challenges they face as they obtain training from the same environment. In this regard, more holistic training on handling field experiences and academic stress‐coping capabilities should be rolled out for the pre‐service teachers.

### 5.1 Practical Implications

The findings have implications for the integration of mental health and wellness programmes into the teacher training process to promote positive well‐being among pre‐service teachers, as this directly relates to improved academic achievements. Universities or teacher preparation institutions should offer a comprehensive support mechanism consisting of professional counseling services, which shall include stress management workshops and resilience training programmes targeting pre‐service teachers. This approach addresses not only the emotional trials in the profession of teaching but also creates a setting where academic excellence thrives. It is also important to stress that the development of ASE should be a priority in the training of teachers by focusing on activities such as goal‐setting exercises, peer mentoring, and reflective teaching practices, which enhance self‐efficacy. These initiatives can help build confidence in teaching abilities, which is crucial for academic success.

While the moderating role of EI may not be significant in the relationship between well‐being and academic success, it still plays a supportive role in handling stress in teacher training. Therefore, development activities of EI, such as emotional awareness training and communication skill‐building, would help prepare pre‐service teachers for effective coping with the emotional demands of their training and subsequently, in the teaching profession. Addressing these areas—mental health supports, ASE development, training in EI, and cultural considerations—the Ghanaian teacher training institutions can then significantly improve the pre‐service teachers’ academic training, well‐being, and professional development.

## 6 Limitations

Despite these contributions to understanding the dynamics among these variables, this study has some limitations. The cross‐sectional design restricts any causal inferences; hence, longitudinal studies will be necessary to establish directionality among the constructs. Moreover, self‐report measures may have the effect of response bias, where respondents may overestimate their well‐being or self‐efficacy level. The sample size of 660 participants may not be representative of the broader population, particularly as this study focused on the University of Education, Winneba, Ghana. This limits the generalizability of the findings to other populations or settings, such as different cultural backgrounds or educational systems (Dzakadzie and Quansah 2023). Hence, any interpretation or use of the findings must be done considering the sample and population.

## 7 Conclusions

In conclusion, EI is helpful in academic excellence in pre‐service teacher training, but its interaction with PWB and ASE is not without more exploitation. The study findings highlight the critical role played by EI and ASE in the training process of teachers, fostering academic excellence while they navigate the challenges in their training. Although all variables were relevant in explaining the variations in the academic achievements of pre‐service teachers, ASE had the strongest role to play in terms of impacting their training success. Understanding these dynamics will help educators and curriculum developers integrate into pre‐service teachers’ training elements of ASE and EI to enhance their professional and academic development.

## Author Contributions

**Frank Quansah**: conceptualization, methodology, software, data curation, validation, investigation, formal analysis, writing – original draft, writing – review and editing. **Daniel William Essel**: conceptualization, data curation, formal analysis, investigation, visualization, validation, writing – original draft, writing – review and editing. **Nancy Asieduwaa Gyapong**: conceptualization, methodology, data curation, investigation, visualization, writing – original draft, writing – review and editing. **Isaac N‐Nandi Yabana**: conceptualization, methodology, investigation, writing – original draft, writing – review and editing. **Yayra Dzakadzie**: data curation, investigation, methodology, validation, visualization, writing – original draft, writing – review and editing. **Patrick Asamoah**: methodology, investigation, writing – review and editing. **Kingsley Addison**: investigation, conceptualization, validation, writing – original draft.

### Peer Review

The peer review history for this article is available at https://publons.com/publon/10.1002/brb3.70578

## Data Availability

The data that support the findings of this study are available from the corresponding author upon reasonable request.
